# The epidemiology of intensive care unit-acquired hyponatraemia and hypernatraemia in medical-surgical intensive care units

**DOI:** 10.1186/cc7162

**Published:** 2008-12-18

**Authors:** Henry Thomas Stelfox, Sofia B Ahmed, Farah Khandwala, David Zygun, Reza Shahpori, Kevin Laupland

**Affiliations:** 1 Department of Critical Care Medicine, University of Calgary, Foothills Medical Centre, EG23, 1403-29 Street NW, Calgary, AB T2N 2T9, Canada; 2 Department of Community Health Sciences, University of Calgary, Calgary, AB T2N 2T9, Canada; 3 Department of Medicine, University of Calgary, Calgary, AB T2N 2T9, Canada; 4 Alberta Kidney Disease Network, Calgary, AB T2N 2T9, Canada; 5 Calgary Health Region Research Portfolio, Calgary Health Region, Rm 1103, 1403-29 Street NW, Calgary, AB T2N 2T9, Canada; 6 Department of Clinical Neurosciences, University of Calgary, Foothills Medical Centre, EG23, 1403-29 Street NW, Calgary, AB T2N 2T9, Canada

## Abstract

**Introduction:**

Although sodium disturbances are common in hospitalised patients, few studies have specifically investigated the epidemiology of sodium disturbances in the intensive care unit (ICU). The objectives of this study were to describe the incidence of ICU-acquired hyponatraemia and hypernatraemia and assess their effects on outcome in the ICU.

**Methods:**

We identified 8142 consecutive adults (18 years of age or older) admitted to three medical-surgical ICUs between 1 January 2000 and 31 December 2006 who were documented to have normal serum sodium levels (133 to 145 mmol/L) during the first day of ICU admission. ICU acquired hyponatraemia and hypernatraemia were respectively defined as a change in serum sodium concentration to below 133 mmol/L or above 145 mmol/L following day one in the ICU.

**Results:**

A first episode of ICU-acquired hyponatraemia developed in 917 (11%) patients and hypernatraemia in 2157 (26%) patients with an incidence density of 3.1 and 7.4 per 100 days of ICU admission, respectively, during 29,142 ICU admission days. The incidence of both ICU-acquired hyponatraemia (age, admission diagnosis, Acute Physiology and Chronic Health Evaluation (APACHE) II score, length of ICU stay, level of consciousness, serum glucose level, body temperature, serum potassium level) and ICU-acquired hypernatraemia (baseline creatinine, APACHE II score, mechanical ventilation, length of ICU stay, body temperature, serum potassium level, level of care) varied according to patients' characteristics. Compared with patients with normal serum sodium levels, hospital mortality was increased in patients with ICU-acquired hyponatraemia (16% versus 28%, p < 0.001) and ICU-acquired hypernatraemia (16% versus 34%, p < 0.001).

**Conclusions:**

ICU-acquired hyponatraemia and hypernatraemia are common in critically ill patients and are associated with increased risk of hospital mortality.

## Introduction

Sodium disturbances, leading to hyponatraemia and hypernatraemia, are a common problem in adult patients admitted to hospital and are associated with hospital mortality rates ranging from 42% to 60% [1-7]. Because of their incapacitation, lack of free access to water and the usually serious nature of their underlying diseases, patients in the intensive care unit (ICU) are at high risk of developing sodium disturbances [8]. However, previous studies suggest that sodium disturbances that are acquired in the hospital are largely preventable [9,10]. Patients in the ICU are well monitored and blood samples are taken frequently. Furthermore, the maintenance of fluid and electrolyte balance is one of the focal points of critical care. Therefore, swift adaptations in fluid and electrolyte administration would be expected to be implemented in situations in which the development of a sodium disturbance might be expected or if a disturbance was detected.

However, the epidemiology of sodium disturbances in critically ill patients has not been well defined. In a retrospective one-year study from a Dutch Medical ICU, Polderman and colleagues reported hypernatraemia (defined as a sodium level of 150 mmol/L or higher) in 9% of patients admitted to the ICU with an additional 6% of patients developing hypernatraemia during their ICU stay [11]. Patients who presented with hypernatraemia had a 20% hospital mortality rate compared with 32% in patients who acquired hypernatraemia during their ICU stay [11]. Lindner and colleagues described a similar incidence of hypernatraemia in a medical ICU in Austria, but reported higher hospital mortality rates in patients presenting with hypernatraemia than in those acquiring the disorder (43% versus 39%) [12]. Similarly, in a retrospective five-year review of a medical ICU in France, Bennani and colleagues reported a 14% incidence of hyponatraemia (defined as a sodium level below 130 mmol/L), with severe hyponatraemia (defined as a sodium level below 125 mmol/L) being associated with increased mortality [13].

Although these three studies are important contributions to the literature, further study is needed to better define the epidemiology of ICU-acquired sodium disturbances. Results from the three studies may not be widely applicable to critically ill populations because of their limited sample size [11], focus on medical patients such that the epidemiology of sodium disturbances in a critically ill surgical patient is unknown [11-13] and exclusive reporting from single ICUs in tertiary care referral hospitals [11,13]. We therefore undertook a study of patients admitted to three medical-surgical ICUs to describe the incidence of ICU-acquired hyponatraemia and hypernatraemia and assess their effects on outcome among a large cohort of adults admitted to all ICUs in a large Canadian health region.

## Materials and methods

### Study population

The Calgary Health Region (CHR) administers all publicly funded hospital care to the residents of the city of Calgary and surrounding areas (population 1.2 million) in the province of Alberta, Canada [14]. All critically ill adult patients in the CHR are managed in ICUs under the care of the Department of Critical Care Medicine. These ICUs are closed units, staffed by fully trained intensivists and currently include one 24-bed medical-surgical ICU that serves as the regional neurosurgical and trauma referral centre: one 14-bed medical-surgical ICU that is also the vascular surgery referral centre; and a 10-bed medical-surgical ICU.

For this study, we utilised a population-based inception cohort design. We identified consecutive adults (18 years of age or older) admitted to the three medical-surgical ICUs in the CHR between 1 January 2000 and 31 December 2006. Patients with more than one admission to the ICU during the study period only had their first ICU admission selected for review. Patients were included in the study cohort if their ICU stay was longer than one day in duration and they were documented to have exclusively 'normal' serum sodium level(s) as per the Calgary Laboratory Services (CLS) reference range (133 to 145 mmol/L) during the first day of their ICU admission. Patients who received renal replacement therapy during their ICU admission were excluded. The Conjoint Health Research Ethics Board at the University of Calgary and CHR approved this study and waiver of patient consent.

### Data sources

Demographic, hospital and clinical data were obtained using the regional ICU patient data warehouse. Data sources include an electronic patient information system (Quantitative Sentinel; GE-Marquette Medical Systems Inc, Milwaukee, WI, USA) that is interfaced to all bedside monitoring and ventilator devices that capture physiological and ventilation data. These data were validated by nursing or respiratory therapy staff on at least an hourly basis by examining the degree to which they are representative and plausible. An HL-7 interface with the regional laboratory information system (Cerner PathNet Classic version 306, Kansas City, MO, USA) was utilised to collect laboratory data. The most abnormal (maximum and minimum) physiological and laboratory values in each 24-hour period (00:00 hours to 23:59 hours) were exported to the data warehouse. For analysis purposes, the value that deviated the furthest from the median of the reference range was taken. Where there was no difference between the minimum and maximum value from the median, the maximum value was taken. A sensitivity analysis was performed using the minimum value and produced similar results.

### Patient characteristics

Patient characteristics were classified *a priori *into time-independent factors and time-dependent factors. Time-independent factors included demographic (age, sex), hospital (admission location, admission ICU, weekend admission, night admission), clinical (admission diagnosis, admission Acute Physiology and Chronic Health Evaluation (APACHE) II score, admission Therapeutic Intervention Scoring System (TISS) score) characteristics. Time-dependent patient factors included vital signs, Glasgow Coma Score, all laboratory values and level of care (full care, full care without cardiopulmonary resuscitation (CPR), comfort care). Severity of illness at inception (within the first day of ICU admission) was assessed using the APACHE II score and intensity of care using the TISS score [15,16].

Patients were classified into three categories of admission diagnosis, based on data recorded by the admitting physician, medical, surgical or neurological/trauma. Hyponatraemia was defined as a serum sodium concentration less than 133 mmol/L. Hypernatraemia was defined as a serum sodium concentration greater than 145 mmol/L. Patients were classified as experiencing multiple distinct sodium disturbances if abnormal serum sodium measurements were separated by a minimum of one day of normal serum sodium measurements. Patients with more than one distinct sodium disturbance only had their first episode of ICU-acquired hyponatraemia or hypernatraemia selected to describe the incidence of sodium disturbances. Baseline renal dysfunction was defined as a creatinine level greater than 100 μmol/L during the first day of ICU admission (CLS reference range less than 100 μmol/L for adult females). A normal core body temperature was defined as 35.0 to 37.3°C [17]. A normal serum concentration of potassium was defined as 3.5 to 5.0 mmol/L.

### Statistical analysis

Data were initially summarised with the mean, median, standard deviations and interquartile ranges for continuous variables and frequencies for categorical variables. In order to make univariable comparisons between normal, hyponatraemic and hypernatraemic subgroups, chi-squared tests were used for categorical variables and analysis of variance was used for continuous variables. Missing laboratory values were imputed with the value on the closest previous or following day where available, within a 48-hour window. Multivariable models for acquiring hyponatraemia and hypernatraemia were determined using generalised estimating equations with a logistic regression in order to adjust for repeated measures. A first-order autoregressive correlation structure was assumed for both models because of the longitudinal nature of the data. Outcome models were formulated using logistic regression. For each model, backward selection was used to find the most parsimonious model. All results were calculated using SAS (version 9.1) and a significance level of 0.05 was used for all analyses.

## Results

### Baseline data

During the seven-year study period, 12,744 adults were admitted to the three medical-surgical ICUs, of which 8142 (64%) were documented to have normal serum sodium levels during their first day of ICU admission and an ICU stay greater than one day. The baseline characteristics of the study population (n = 8142) are summarised in Table 1. Forty-one percent (n = 3323) of patients were female, the median age was 59.7 years (interquartile range (IQR) = 43.2 to 73.4 years), and the mean APACHE II score at first admission was 18.5 (standard deviation [SD] = 7.9). Of the ICU admissions, 3574 (44%) were classified as medical, 2395 (30%) as surgical and 2142 (26%) as neurological/trauma. The mean serum sodium value for patients during their first day of ICU admission was 139.1 mmol/L (SD = 3.5 mmol/L).

**Table 1:** Characteristics of patients with normal serum sodium on day one in the intensive care unit (ICU)*^†^

	**Serum Sodium Category**		
	
**Characteristics**	**Acquire hyponatraemia** **(n = 917)**	**Always normal** **(n = 5068)**	**Acquire hypernatraemia** **(n = 2157)**
**Demographic**			
Age, mean, years	57 (19)	56 (20)	60 (18)
Female, number (%)	397 (43)	2060 (41)	866 (40)
**Hospital**			
Admission location, number (%)			
Emergency department	337 (37)	1926 (38)	833 (39)
Operating room	243 (27)	1450 (29)	509 (24)
Hospital floor	217 (24)	1049 (21)	534 (25)
Transfer from another facility	118 (13)	632 (13)	280 (13)
Admission ICU, number (%)			
Trauma and neurosurgery referral ICU	534 (58)	2568 (51)	1127 (52)
Vascular surgery referral ICU	214 (23)	1388 (27)	595 (28)
General medical-surgical ICU	169 (18)	1112 (22)	435 (20)
Weekend admission, number (%)	265 (29)	1294 (26)	599 (28)
Night admission, number (%)	536 (58)	2787 (55)	1267 (59)

**Clinical**			
Admitting diagnosis category, number (%)			
Neurological/trauma	256 (28)	1279 (25)	607 (28)
Surgical	255 (28)	1570 (31)	570 (26)
Medical	404 (44)	2191 (43)	979 (45)
Vasoactive medication first 24 hours, number (%)	318 (35)	936 (19)	820 (38)
Mechanical ventilation first 24 hours, number (%)	619 (67)	3277 (65)	1629 (75)
Temperature, °C	37.0 (1.5)	37.0 (1.3)	36.8 (1.6)
Glasgow Coma Scale score	9.2 (4.4)	9.6 (4.3)	8.1 (4.3)
Serum sodium, mmol/L	137 (3)	139 (3)	140 (3)
Serum potassium, mmol/L	4.0 (0.9)	3.9 (0.7)	4.0 (0.9)
Serum glucose, mmol/L	9.2 (4.0)	8.9 (3.7)	10.0 (4.9)
Serum creatinine, median (IQR) μmol/L	78 (56 to 133)	76 (59 to 104)	90 (64 to 143)
APACHE II Score	19.8 (7.9)	16.9 (7.5)	21.8 (7.7)
TISS score	39.1 (13.2)	32.3 (11.9)	40.8 (12.5)
Level of care, number (%)			
Full care	887 (97)	4799 (95)	2018 (94)
Full care, but no CPR	30 (3)	258 (5)	135 (6)
Comfort care	0 (0)	11(0)	4 (0)

*Results reported as mean (standard deviation) unless indicated.

†Physiological and laboratory data represent the most abnormal values recorded during the first day in ICU.

APACHE = Acute Acute Physiology and Chronic Health Evaluation, CPR = cardiopulmonary resuscitation, IQR = interquartile range, TISS = Therapeutic Intervention Scoring System.

### Incidence

**Figure 1:**
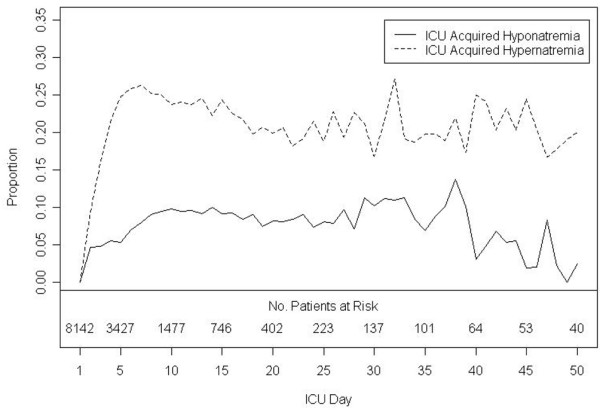
Proportion of intensive care unit (ICU) patients with serum sodium values outside the normal range during the first 50 days of ICU stay*.

Among the 8142 patients with normal serum sodium levels during their first day of ICU admission, a first episode of ICU acquired hyponatraemia developed in 917 (11%) patients and hypernatraemia in 2157 (26%) patients. Among a total of 29,142 ICU admission days, the incidence density for a first episode of ICU-acquired hyponatraemia and hypernatraemia were 3.1 and 7.4 per 100 days of ICU admission, respectively (Figure 1). The median time from ICU admission to patients developing an ICU-acquired sodium disturbance was two days for both hyponatraemia (IQR = one to five days) and hypernatraemia (IQR = one to three days). Twenty five percent of the patients with a sodium disturbance experienced more than one distinct sodium disturbance during their ICU stay. Sixteen percent (n = 150) of patients with ICU-acquired hyponatraemia experienced more than one episode of hyponatraemia compared with 19% (n = 413) of patients with ICU-acquired hypernatraemia who experienced more than one episode of hypernatraemia (p = 0.067). Distinct episodes of both hyponatraemia and hypernatraemia were experienced by 196 patients (6.4% of patients with ICU-acquired sodium disturbances) during their ICU stay. The mean serum sodium levels for patients during episodes of ICU-acquired hyponatraemia and hypernatraemia were 130 mmol/L (SD = 2.7 mmol/L) and 149 mmol/L (SD = 3.6 mmol/L), respectively. Among patients with sodium disturbances, the median number of days of hyponatraemia (IQR = one to three days) and hypernatraemia (IQR = one to five days) was two.

### Multivariable analysis of patient characteristics

The incidence of ICU-acquired hyponatraemia and hypernatraemia varied according to patient characteristics (Table 2). Higher APACHE II scores, longer ICU stays as well as body temperature disturbances (hypothermia or fever) were associated with both ICU-acquired hyponatraemia and hypernatraemia. Serum potassium disturbances had an inverse relationship with sodium disturbances. Hyperkalaemia was associated with ICU-acquired hyponatraemia, while hypokalaemia was associated with ICU-acquired hypernatraemia. Age, neurological/trauma or surgical admitting diagnosis, level of consciousness and serum glucose were additional factors associated with ICU-acquired hyponatraemia, while baseline creatinine, mechanical ventilation and level of care were associated with ICU-acquired hypernatraemia.

**Table 2:** Multivariable analyses of patient characteristics*

	**Acquire hyponatraemia**		**Acquire hypernatraemia**	
		
**Characteristic**	**Odds ratio (95% CI)**	**P Value**	**Odds ratio (95% CI)**	**P value**
Age (for each 10 year increase)	0.93 (0.89 to 0.98)	0.004	NS	NS
Baseline creatinine >100 μmol/L	NS	NS	1.47 (1.31 to 1.65)	<0.001
Admitting diagnosis category			NS	NS
Medical	1.00			
Neurological/trauma	1.33 (1.06 to 1.65)	0.012		
Surgical	1.26 (1.04 to 1.52)	0.017		
APACHE II score (for each additional unit)	1.08 (1.06 to 1.09)	<0.001	1.05 (1.04 to 1.05)	<0.001
Mechanical ventilation	NS	NS	1.30 (1.20 to 1.42)	<0.001
Day of ICU stay (for each additional log unit day^‡^)	1.95 (1.81 to 2.10)	<0.001	2.06 (1.95 to 2.17)	<0.001
Minimum Glasgow Coma Scale (for each additional unit)	1.06 (1.03 to 1.08)	<0.001	NS	NS
Glucose level (for each additional 1 mmol/L)	1.07 (1.06 to 1.09)	<0.001	NS	NS
Temperature				
35.0 to 37.3°C^†^	1.00		1.00	
>37.3°C	1.36 (1.10 to 1.69)	0.005	1.30 (1.16 to 1.45)	<0.001
<35.0°C	1.36 (1.08 to 1.70)	0.008	1.28 (1.14 to 1.44)	<0.001
Serum potassium				
3.5 to 5.0 mmol/L^†^	1.00		1.00	
>5.0 mmol/L	1.67 (1.42 to 1.97)	<0.001	1.05 (0.93 to 1.19)	0.421
<3.5 mmol/L	1.01 (0.90 to 1.14)	0.846	1.49 (1.40 to 1.59)	<0.001
Level of care	NS	NS		
Full care			1.00	
Full care, but no CPR			1.23 (1.09 to 1.39)	0.001
Comfort care			1.35 (1.07 to 1.70)	0.010

*Time-independent (age, baseline creatinine, random glucose) and time-dependent (minimum Glasgow coma scale, glucose level, Acute Acute Physiology and Chronic Health Evaluation (APACHE) II score, mechanical ventilation, day of intensive care unit (ICU) stay, temperature, serum potassium, level of care) characteristics included in multivariable models.

†Patients with this factor served as the reference group

‡Length of ICU stay was highly skewed and time unit day was log transformed.

CI = confidence interval, CPR = cardiopulmonary resuscitation, NS = not significant.

### Outcomes of care

**Figure 2:**
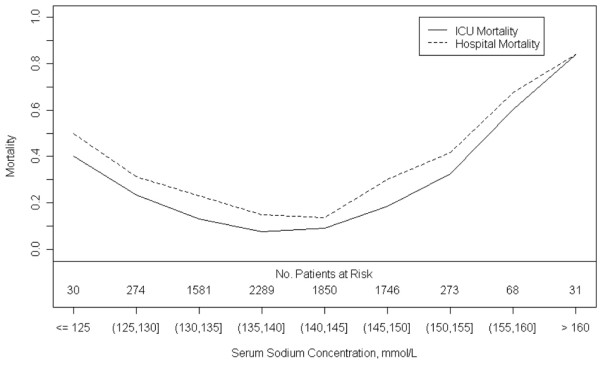
**Maximum deviation of serum sodium level from normal range during intensive care unit (ICU) admission and patient mortality**.

Length of stay and mortality in the ICU and hospital were increased for patients with ICU-acquired hyponatraemia and hypernatraemia compared with patients with normal serum sodium levels (Table 3). Similar outcomes of care were observed for patients with medical, surgical and neurological/trauma diagnoses. A dose response relationship was observed for the magnitude of the ICU-acquired sodium disturbance (absolute deviation from normal range) and both ICU (p < 0.001) and hospital mortality (p < 0.001) (Figure 2). The duration of ICU-acquired sodium disturbances and the daily rate of change in serum sodium levels were both associated with ICU and hospital mortality, but provided no significant explanatory power above the magnitude of the sodium disturbance.

**Table 3:** Outcomes of care

	**Serum sodium category**			
		
**Measures**	**Acquire hyponatraemia** **(n = 917)**	**Always normal** **(n = 5068)**	**Acquire hypernatraemia** **(n = 2157)**	**p value**^‡^
ICU length of stay, median (IQR), d^†^	6 (3 to 12)	2 (1 to 4)	7 (4 to 13)	<0.001
Hospital length of stay, median (IQR), d^†^	25 (14 to 50)	12 (7 to 24)	24 (14 to 51)	<0.001
ICU mortality, number (%)	164 (18)	456 (9)	488 (23)	<0.001
Hospital mortality, number (%)	255 (28)	799 (16)	723 (34)	<0.001

†ICU and hospital length of stay for patients who survive to hospital discharge.

‡p values calculated by multivariable linear and logistic regression for comparisons of patients who acquire hyponatraemia or hypernatraemia with patients who always have normal serum sodium levels.

ICU = intensive care unit, IQR = interquartile range.

## Discussion

Our study is the first multi-centred evaluation of ICU-acquired sodium disturbances in a non-select population of medical-surgical critically ill patients. It is also the first study to attempt to characterise the longitudinal nature of sodium disturbances with a time-dependent data set. The results demonstrate that ICU-acquired hyponatraemia and hypernatraemia are common in critically ill patients. The occurrence of ICU-acquired hyponatraemia and hypernatraemia varies significantly among patients with different demographic and clinical characteristics. There is a strong association between both ICU-acquired hyponatraemia and hypernatraemia and in-hospital patient mortality.

Our study provides three important contributions to the epidemiology of sodium disturbances in critically ill patients in addition to the previously published works by Polderman and colleagues [11], Lindner and colleagues [12] and Bennani and colleagues [13]. First, our study extends the general applicability of the literature to a broader population of critically ill patients because we examined a non-select population of patients with medical, surgical and neurological/trauma diagnoses as compared with the previous studies that focused only on patients in medical ICUs.

Second, we examined both ICU-acquired hyponatraemia and hypernatraemia in our study, while the previous works focused respectively on a single disturbance. This allowed us to make the observation that ICU-acquired hypernatraemia has twice the incidence of hyponatraemia and that patients with surgical and neurological/trauma diagnoses are at increased risk of developing hyponatraemia compared with medical patients, but at similar risk of hypernatraemia.

Third, we identified several patient characteristics that were associated with ICU-acquired sodium disturbances, and could potentially be used to help clinicians identify patients at increased risk. An elevated baseline creatinine was associated with a 50% increased risk of ICU-acquired hypernatraemia and may be a marker of impaired renal sodium and water regulation or decreased intravascular volume [18]. Mechanical ventilation was associated with ICU-acquired hypernatraemia. Mechanical ventilation may be a marker of illness severity, but it also inhibits patient-clinician communication and makes patients dependent on others for their water requirements [19]. Length of stay in the ICU was associated with both ICU-acquired hyponatraemia and hypernatraemia. This relationship is likely to reflect multiple risk factors including increased illness severity for patients with long ICU stays, an increased exposure period to adverse events and clinician distraction as patients become chronically critically ill [20,21].

Finally, increasing APACHE II scores were associated with both ICU-acquired hyponatraemia and hypernatraemia. All of these observations raise the question of whether sodium disturbances are a physiological disturbance that independently increases the risk of death, a marker of illness severity or both. Serum sodium levels have been incorporated into validated illness severity scores such as the APACHE II score [15]. However, in our analyses, even after adjusting for patients' characteristics including renal function, mechanical ventilation and APACHE II scores, ICU-acquired sodium disturbances were independently associated with mortality.

Our study underscores the challenges to improve management of ICU-acquired sodium disturbances. Previous studies have suggested that the majority of sodium disturbances acquired in hospital are preventable and indicative of substandard care [9,10]. Sodium disturbances in the ICU according to our study appear to develop insidiously, present a median of two days after admission and with moderate deviations from the normal range (mean hyponatraemia = 130 mmol/L, mean hypernatraemia = 149 mmol/L). Identifying these disturbances may be difficult for clinicians preoccupied with more acute medical issues or other laboratory investigations. For example, in our study the mean number of laboratory tests performed on patients in the ICU ranged from 61 to 74 individual laboratory tests per patient per day and it can therefore be surmised that a single abnormal serum sodium level may be lost in this sea of laboratory values.

Developing strategies to prevent or correct ICU-acquired sodium disturbances are also more challenging than it first appears. An important and novel finding of our study is that a strong association exists between the magnitude of ICU-acquired sodium disturbances and hospital mortality. The dose-response relation between sodium deviation and hospital mortality highlights that even small deviations in serum sodium concentration from the normal range are associated with increased mortality. Physicians regulate the water and electrolyte balance in most patients in the ICU, therefore augmenting the risk of iatrogenic electrolyte derangements. The most effective way to reduce this risk is to allow patients to resume control and regulation of their own fluid and electrolyte balance as soon as it is safely possible. Studies are needed to establish optimal strategies for monitoring, diagnosing and managing ICU-acquired sodium disturbances.

The results of our study need to be interpreted within the context of its limitations. First, our data are based on a clinical data source that captures detailed demographic, hospital, physiological and laboratory data, but limited information on interventions. For example, intravenous fluids, nutrition (enteral and parental), fluid balance and medications (e.g. osmotic therapy) were not reliably captured in our data source and were therefore excluded from the analyses. As such, it is difficult to determine both the aetiology of the ICU-acquired hyponatraemia and hypernatraemia and clinicians' responses. Second, our study was observational in nature and designed to describe the epidemiology of sodium disturbances in a population of critically ill patients. As such our observations are valuable for generating hypotheses, but not causal inference. Third, our results are based on patients admitted to three medical-surgical ICUs in a single health region. Although our data are population based and reflect the management of all patients admitted to ICUs under the care of 26 intensive care specialists, it is possible that patients treated in other types of ICUs or in other health regions or with other diagnoses may have different experiences.

## Conclusions

In summary, this large study conducted in a broad non-select population of adult patients admitted to ICUs demonstrates that ICU-acquired hyponatraemia and hypernatraemia are common in the critically ill. The risk of ICU-acquired sodium disturbances appear to vary according to patient characteristics. Finally, ICU-acquired hyponatraemia and hypernatraemia are associated with increased in-hospital mortality. Studies are needed to establish optimal management strategies.

## Key messages

• ICU-acquired hyponatraemia and hypernatraemia develop in up to one-quarter of critically ill patients with hypernatraemia being twice as common as hyponatraemia.

• The incidence of ICU-acquired hyponatraemia and hypernatraemia varies according to patient characteristics.

• ICU-acquired hyponatraemia and hypernatraemia are associated with increased risk of hospital mortality – a dose-response relation appears to exist for the magnitude of the ICU-acquired sodium disturbance.

## Abbreviations

APACHE: Acute Physiology And Chronic Health Evaluation; CHR: Calgary Health Region; CLS: Calgary Laboratory Services; CPR: cardiopulmonary resuscitation; ICU: intensive care unit; IQR: interquartile range; SD: standard deviation; TISS: Therapeutic Intervention Scoring System.

## Competing interests

The authors declare that they have no competing interests.

## Authors' contributions

HTS designed the study, acquired data, interpreted data, drafted and revised the manuscript. SBA interpreted data, drafted and revised the manuscript. FK analysed and interpreted data and drafted the manuscript. DZ interpreted data and revised the manuscript. RS acquired data and revised the manuscript. KL acquired data, interpreted data and revised the manuscript. HTS and FK had full access to all the study data and assume responsibility for the integrity of the data and the accuracy of the analysis.
